# No age effect in the prevalence and clinical significance of ultra-high risk symptoms and criteria for psychosis in 22q11 deletion syndrome: Confirmation of the genetically driven risk for psychosis?

**DOI:** 10.1371/journal.pone.0174797

**Published:** 2017-04-13

**Authors:** Marco Armando, Maude Schneider, Maria Pontillo, Stefano Vicari, Martin Debbané, Frauke Schultze-Lutter, Stephan Eliez

**Affiliations:** 1 Child and Adolescence Neuropsychiatry Unit, Department of Neuroscience, Children Hospital Bambino Gesù, Rome, Italy; 2 Developmental Imaging and Psychopathology Lab, Department of Psychiatry, School of Medicine, University of Geneva, Geneva, Switzerland; 3 University Hospital of Child and Adolescent Psychiatry and Psychotherapy, University of Bern, Bern, Switzerland; Maastricht University, NETHERLANDS

## Abstract

**Background:**

The 22q11.2 deletion syndrome (22q11DS) is one of the highest known risk factors for schizophrenia. Thus, the detection of 22q11DS patients at particularly high risk of psychosis is important, yet studies on the clinical significance of the widely used ultra-high risk (UHR) criteria in 22q11DS are inconclusive. Since age was reported to moderate clinical significance of UHR symptoms in community samples, we explored whether age at presentation of UHR symptoms and criteria may explain part of this heterogeneity.

**Methods:**

111 patients with 22q11DS (8–30 years; 15.7±4.7) were assessed for UHR symptoms/criteria. Information on diagnoses, psychosocial functioning, and IQ were collected.

**Results:**

Any UHR symptom was reported by 38.7%, any UHR criterion by 27%. No significant influence of age on the prevalence of UHR symptoms or criteria was detected. Moreover, age did not significantly modulate the association between UHR symptoms and functioning. However, significant interaction terms suggested that younger age groups were more likely to meet UHR criteria in the presence of UHR symptoms compared to the adult group.

**Discussion:**

Compared to the general population, prevalence of UHR symptoms and criteria was 3.8-fold and 20.8-fold in our 22q11DS sample. Contrary to the general population, age only modulated the prevalence of UHR criteria among those with UHR symptoms, but not their prevalence per se or their clinical significance. This suggests that UHR symptoms might develop as a trait factor in terms of a genetically driven schizotypal disposition in 22q11DS, thus necessitating future studies on psychosis-risk indicators in this genetic high-risk group.

## 1. Introduction

The 22q11.2 deletion syndrome (22q11DS) is a genetic condition characterized by a microdeletion of 3 million base pairs of DNA on chromosome 22 band q11. It occurs at an estimated prevalence of 2000–4000 live births [1], and is currently recognized as one of the highest known risk factors for schizophrenia [2]. While 23% to 45% of adolescents with 22q11DS report transient psychotic experiences [3–6], up to 40% of affected adults are diagnosed with a psychotic disorder [7]. Moreover, 22q11DS was found in 0.3% to 2.0% of patients with schizophrenia [8–10] and up to 5.7% of patients with early-onset schizophrenia [11], suggesting that 22q11DS is often characterized by an early onset of psychosis. Consistently, a longitudinal study observed a mean age of onset of 17.7 years in patients with 22q11DS who developed a psychotic disorder [12], on average two years earlier than in the general population. Despite this difference, the clinical presentation of schizophrenia in 22q11DS is comparable to that observed in the general population [13], confirming that 22q11DS is a valuable model to unravel mechanisms contributing to psychosis. The detection of patients with 22q11DS at particularly high risk of conversion to psychosis hence appears as an important area of investigation. Two clinical tools were developed to identify individuals at clinical high-risk for psychosis, the ultra high-risk (UHR) and the basic symptom (BS) criteria [14, 15]. Symptomatic UHR criteria refer to the presence of attenuated psychotic symptoms (APS) or brief limited intermittent psychotic symptoms (BLIPS) meeting certain frequency or onset/worsening requirements (see Table 1).

**Table 1:** Description of UHR criteria according to the SIPS.

UHR condition	Symptom criterion	Onset/worsening criteria	Frequency criterion
*Attenuated Psychotic Symptoms (APS)*	Any positive symptom (P1-P5) score between 3–5	Development or increase by 1 point in severity within the past year	Average frequency of at least once per week in the past month
*Brief Limited Intermittent Psychotic Symptoms (BLIPS)*	Any positive symptom (P1-P5) score = 6	Development or increase within the past 3 months	Several minutes a day 4 days/week for 1 month
*Genetic Risk + Functional Decline (GRFD)*	Presence of a genetic risk factor (family history of psychosis; schizotypal personality disorder of person) in combination with a recent significant decline in psychosocial functioning		

Previous studies found higher prevalence of UHR symptoms and criteria in 22q11DS than in the general population, while the symptom pattern and age of onset seem to be rather comparable [16]. However reported numbers differed greatly, with prevalence rates of UHR criteria ranging from 10% to 57% [6, 17–19]. In addition, a recent longitudinal study showed that the presence of an UHR condition at baseline significantly predicted transition to psychosis 2.7 years later in a sample of 89 patients with 22q11DS aged 8 to 30 years [18]. Indeed, 27.3% of participants with a UHR condition at baseline converted to psychosis at follow-up, whereas this percentage was only 4.5% in those not meeting UHR criteria at baseline. This finding provided support for the usefulness of UHR criteria in this specific high-risk population. However, it also showed that 45% of patients with a UHR condition at baseline did not convert to psychosis and were no longer meeting UHR criteria at follow-up, suggesting that the clinical significance of UHR criteria is variable. One reason for this heterogeneity in prevalence and transition rates might be age. A recent study [20] reported an age effect on the prevalence and clinical significance of UHR symptoms and criteria using the Structured Interview for Psychosis-Risk Syndromes (SIPS) [21]. Therein, perceptual APS were more prevalent and non-perceptual APS clinically less significant in children and adolescents of 8 to 15 years compared to participants of 16 to 40 years of age.

The goal of the present study was to explore whether age at assessment explained part of the heterogeneity in prevalence of UHR symptoms and criteria in 22q11DS and was associated with differences in their clinical significance. In line with the study of Schimmelmann et al. [20], we expected that the prevalence of UHR symptoms and criteria would be modulated by age in patients with 22q11DS. However, given the earlier age-of-onset of psychosis and the strong genetic load, we expected the age threshold to be lower compared to the general population. Secondly, we hypothesized that the presence of UHR symptoms would have an impact on functioning that might be further modulated by age.

## 2. Methods

### 2.1 Participants

We included 111 participants (77 from Geneva and 34 from Rome) diagnosed with a genetically confirmed 22q11DS, aged between 8 and 30 years (mean = 15.7, SD = 4.7). Participants with a past or present psychotic disorder were excluded, as the focus of the present study is to better understand symptoms that putatively precede the onset of a full-blown psychotic episode.

Participants from the Geneva cohort were voluntarily recruited though advertisements in patient associations or word of mouth. Participants from the Rome cohort were referred to the Neuroscience Department from the Genetic Clinical Unit of the Children Hospital Bambino Gesù and voluntarily recruited there and through advertisement in patient associations. The present study was approved by the Comission Cantonale d’Ethique de la Recherche (Geneva) and the Comitato Etico dell’ Istituto di Ricerca e Cura a Carattere Scientifico Bambino Gesu (Rome), and written informed consent from the participants and their parents was collected at both sites. Eighty-nine participants were also involved in a previous longitudinal study combining the Geneva and Rome samples [18].

### 2.2 Materials

#### 2.2.1 Clinical assessment

All participants completed the SIPS [21] to assess the presence of UHR symptoms and criteria. The SIPS consists of 19 items assessing four symptom domains: Positive Symptoms (P1-P5; unusual thought content, suspiciousness, grandiosity, perceptual abnormalities, and disorganized communication), negative symptoms (social anhedonia or withdrawal, avolition, decreased expression of emotions, decreased experience of emotions and self, impoverished thinking, deterioration of role functioning), disorganized symptoms (odd appearance and behaviour, bizarre thinking, attention and concentration problems, personal hygiene/social skills), and general symptoms (sleep disorders, dysphoric mood, motor disorders, decreased tolerance to normal stress). Each item is rated on a scale of 1–6, with 6 indicating “severe and psychotic” and 3–5 indicating a symptom in the prodromal range. Non-perceptive (P1, P2, P3, and P5) and perceptive (P4) APS/BLIPS were also distinguished. In the current study, subjects were considered positive for UHR symptoms if they fulfilled the “symptom criteria” listed in Table 1, while they were considered positive for the UHR condition if they fulfilled any “symptom criterion” in addition with both the “onset/worsening” and “frequency” criterion as described in Table 1.

For the global assessment of functioning, the Childhood Global Assessment Scale (CGAS) [22] or the Global Assessment of Functioning (GAF) was used in accordance with patient’s age. For both instruments, the median score of ‘60’ was used to distinguish between low (≤60) and normal (>60) functioning.

Furthermore, the presence of any DSM-IV psychiatric disorder was assessed at both sites using structured clinical interviews. At both sites, the Structured Clinical Interview for Axis I DSM-IV (SCID-I) [23] was administered to adult participants and their parents. For participants below 18 years from the Geneva cohort, parents completed the Diagnostic Interview for Children and Adolescents–Revised (DICA-IV) [24] and diagnoses were confirmed with the participant. Because the DICA does not provide a formal assessment of psychotic disorders, the psychotic disorders supplement of the Schedule for Affective Disorders and Schizophrenia for School-Age Children–Present and Lifetime version (K-SADS-PL) [25] was also administered. In Rome, presence of current mental disorders was assessed using the K-SADS-PL, including the psychotic disorders supplement in children and adolescents below 18 years.

#### 2.2.2 Cognitive assessment

Intellectual functioning in children and adolescents below 18 years was assessed using the Wechsler Intelligence Scale for Children–Third edition (WISC-III) [26]. For the remaining participants, the Wechsler Adult Intelligence Scale–Third edition (WAIS-III) [27] was used. Verbal IQ (VIQ), performance IQ (PIQ) and full-scale IQ (FSIQ) were used as global indicators of intellectual functioning.

### 2.3 Statistical analyses

All statistical analyses were performed using SPSS version 21 and, for comparability, followed the analyses used in earlier community study of an age effect [19]. Frequencies and percentages were compared by chi-square tests, and non-normally distributed interval and ordinal data were evaluated by the Mann-Whitney U tests. Binary logistic regression analyses using “enter” were performed to assess effects of different age groups (8–11; 12–14; 15–17; ≥18) on UHR criteria and each of their requirements (see Table 1). The age group with a peak in the onset of first episode psychosis (≥18 years) served as the reference group. To evaluate the potential additional effects of an age*requirement interaction, both the respective UHR requirements on onset/worsening and frequency of APS and BLIPS, respectively, and their interaction with age were entered as independent variables. The interaction with age was considered as relevant when both backward and forward logistic regression analyses equally selected the interaction term as a predictor. In addition, logistic regression analyses were also used to assess the effects of UHR symptoms and their interaction with age on low psychosocial functioning. The latter was transformed into a dichotomous variable (i.e. ≤60 and >60). Throughout, the goodness-of-fit (GoF) was estimated by the Omnibus test. Furthermore, in all regression analyses minimum of 5 events per predictor variable was observed that was reported to commonly ensure sufficient confidence interval coverage for β1 and the related type I error rate of the test of H0, little bias in the estimate of β1, and thereby sufficient power [28].

## 3 Results

### 3.1 Sample characteristics, prevalence of UHR symptoms and criteria

The total sample consisted 85 (76.6%) children and adolescents (i.e. 8–17 years old) and 26 (23.4%) adults (i.e. ≥18 years old). Compared to the Rome cohort, participants from Geneva had a lower full-scale IQ (t(109) = -5.307, p<0.001), yet, did not differ in terms of age (t(109) = 1.141, p = 0.256) and gender (X^2^(1) = 0.982, p = 0.576) (see S1 Table).

In the total sample, 68 (61.3%) individuals did not present any UHR symptom, while 43 (38.7%) reported at least any one UHR symptom. Of these, 30 (27% of the total sample; 69.8% of the patients with at least any one UHR symptom) fulfilled the requirements for the UHR condition as described in Table 1.

Among patients with UHR symptoms, 2 presented BLIPS (both hallucinations), while 41 reported APS. The prevalence of any perceptive APS/BLIPS was highest (29.7%), followed by 16.2% for any unusual thought content, 16.0% for any persecutory idea, 8.1% for any disorganized communication and 2.7% for any grandiosity, thus raising the overall prevalence of any non-perceptual APS/BLIPS to 25.2%.

No significant difference in terms of age, sex, IQ and any axis I diagnosis was found between patients with and without UHR symptoms. However, patients with UHR symptoms showed significantly lower psychosocial functioning (see Table 2).

**Table 2:** Socio-demographic and clinical characteristics of subjects with and without Ultra-High-Risk (UHR) symptoms.

	≥1 UHR symptom	No UHR symptom	Total	Statistics
	(N = 43; 38.7%)	(N = 68; 61.3%)	(N = 111; 100%)	
Male, n (%)	22 (51.2)	30 (44.1)	52 (46.8)	χ^2^_(1)_ = 0.525, *p* = 0.559
Age, Mdn (quartiles)	15.30 (12.92–18.47)	15.07 (12.01–17.03)	15.14 (12.5–17.5)	U = 1587.0, *p* = 0.449
Age group, n (%):				χ2 (3) = 3.022, p = 0.388
8–11 years	6 (27.3)	16 (72.7)	22 (19.8)	
12–14 years	14 (46.7)	16 (53.3)	30 (27.0)	
15–17 years	11 (33.3)	22 (66.7)	33 (29.7)	
≥18 years	12 (46.2)	14 (53.8)	26 (23.4)	
Any current axis I diagnosis, n (%)	31 (72.1)	42 (61.8)	73 (65.8)	χ^2^_(1)_ = 1.248, *p* = 0.308
IQ, mean (SD)	73.6 (11.3)	76.1 (12.7)	75.1 (12.6)	F _(1)_ = 1.044, *p* = 0.309
C-GAS/GAF ≤60, n (%)	31 (72.1)	27 (39.7)	58 (52.3)	χ^2^_(1)_ = 11.075, *p* = 0.001

Thirty persons (27%) fulfilled all UHR requirements. Among these, 22 (73%) fulfilled APS criteria, 3 (13%) BLIPS criteria (1 also met APS criteria), and 11 (37%) the Genetic Risk and Functional Decline (GRFD) criteria (4 also met APS and 1 BLIPS criteria). In this sample, all the participants diagnosed with the GRFD condition met criteria for a schizotypal personality disorder (criterion a) and experienced at least a 30% drop in GAF score over the last month as compared to 12 months ago. None of them had a first degree relative diagnosed with a psychotic disorder.

### 3.2 Age effect on UHR symptoms

Using binary logistic regression analysis, no association between age group and prevalence of any UHR symptom was observed (GoF: χ^2^_(3)_ = 3.063, p = 0.382) (see Table 3). The results remained unchanged when the analyses were run separately for perceptual (GoF: χ^2^_(3)_ = 3.949, p = 0.267) and non-perceptual (GoF: χ^2^_(3)_ = 2.295, p = 0.514) UHR symptoms (see Table 3).

**Table 3:** Effect of age on UHR symptoms prevalence (irrespective of other UHR requirements*).

Age-range	ß	SE	Wald	p	Exp(ß)	95% CIs (Exp(ß))
Any SIPS-P item with score of 3–6						
8–11 yrs	-0.847	0.603	1.977	0.160	0.429	0.132–1.396
12–14 yrs	0.560	0.520	1.159	0.282	1.750	0.632–4.848
15–17 yrs	-0.539	0.540	0.998	0.318	0.583	0.203–1.680
Any SIPS-P non-perceptive item with score of 3–6						
8–11 yrs	1.035	0.753	1.891	0.169	2.815	0.644–12.306
12–14 yrs	0.201	0.592	0.115	0.735	1.222	0.383–3.903
15–17 yrs	0.170	0.577	0.087	0.769	1.185	0.382–3.675
SIPS-P perceptive item with score of 3–6						
8–11 yrs	0.511	0.626	0.666	0.414	1.667	0.489–5.683
12–14 yrs	0.077	0.553	0.019	0.890	1.080	0.365–3.193
15–17 yrs	1.034	0.605	2.920	0.087	2.812	0.859–9.209

Binary logistic regression analyses with method “enter” and ≥18-year-olds as reference age group.

* See Table 1 for a description of the UHR requirements

No distinctions were found in term of age group when only fully met UHR criteria were considered (GoF: χ^2^_(3)_ = 4.918, p = 0.178) (see Table 4). Again, results remained unchanged when this analysis was run separately for those fulfilling UHR criteria by perceptual (GoF: χ^2^_(3)_ = 5.639, p = 0.131) vs. non-perceptual (GoF: χ^2^_(3)_ = 2.165, p = 0.539) phenomena (see Table 4).

**Table 4:** Effect of age on UHR status (considering all the UHR criteria*).

Age range	ß	SE	Wald	p	Exp(ß)	95% CIs (Exp(ß))
8–11 yrs	-0.588	0.655	0.806	.369	0.556	0.154–2.005
12–14 yrs	0.089	0.560	0.026	.873	1.094	0.365–3.277
15–17 yrs	-1.087	0.637	2.912	.088	0.337	0.097–1.175
UHR status fulfilled by non perceptive phenomena						
8–11 yrs	1.674	1.399	1.431	0.232	5.333	0.343–82.827
12–14 yrs	1.099	1.258	0.762	0.383	3.000	0.255–35.334
15–17 yrs	1.674	1.399	1.431	0.232	5.333	0.343–82.827
UHR status fulfilled by perceptive phenomena						
8–11 yrs	-19.950	17974.843	0.000	0.999	0.000	0.000 –
12–14 yrs	0.272	1.049	0.067	0.796	1.313	0.168–10.264
15–17 yrs	1.658	1.215	1.863	0.172	5.250	0.485–56.80

Binary logistic regression analyses with method “enter” and ≥18-year olds as reference age group.

* See Table 1 for a description of the UHR requirements

### 3.3 Interaction between age and UHR symptoms on the presence of UHR status

Testing for the effect of the interaction of age and presence of any UHR symptom on the presence of any symptomatic UHR criterion with forward and backward stepwise regression analyses, the interaction term predicted UHR status better than the single terms age or presence of any UHR symptom. Leading to a correct classification of 83.8% of cases (95.1% of cases without and 53.3% of cases with UHR criteria), the model became highly significant (Omnibus test: χ^2^_(3)_ = 38.883, p<0.001) and explained 39.2% of the variance (Table 5).

**Table 5:** Result of the stepwise regression analyses of the interaction of age and UHR symptoms on UHR status* (Wald method, forward and backward selection) with “age ≥18 years” and “no UHR symptoms” as reference values.

Predictor	ß	SE	Wald (df)	p	Exp(ß)	95% CIs (Exp(ß))
age group * presence of any UHR symptom			26.445 (3)	<.001		
8–11 years * UHR present	3.555	1.146	9.618 (1)	.002	35.000	3.700–331.059
12–14 years * UHR present	3.245	0.734	19.556 (1)	<.001	25.667	6.091–108.148
15–17 years * UHR present	1.386	0.712	3.789 (1)	.052	4.000	0.991–16.152
constant	-1.946	0.338	33.132 (1)	<.001	0.143	

* See Table 1 for a description of the UHR requirements

Visual inspection of the interaction with age (see S1 Fig) revealed that an UHR status in the presence of an UHR symptom rating became less likely with age, indicating that both onset/worsening and frequency requirements of an UHR status were less likely met in the older age segment of the UHR symptom-positive group. Fourteen participants with UHR symptoms did not meet criteria for a UHR condition for the following reasons: a) onset/worsening and frequency criteria were not met (N = 8; 1 participant in the 8–11 group; 2 participants in the 12–14 group; 2 participants in the 15–17 group; and 3 participants in the ≥18 group); b) frequency criterion was not met (N = 6; 1 participant in the 12–14 group; 5 participants in the 15–17 group).”

### 3.4 Interaction of age and UHR symptoms on level of functioning

In univariate regression analyses, low functioning was predicted by the presence of any UHR symptom (GoF: X^2^_(1)_ = 11.373, p < 0.001), while the effect of age was non-significant (GoF: χ^2^_(1)_ = .752, p = 0.386). When the interaction between age and presence of any UHR symptom was entered in addition to single effects in stepwise regression analyses, the interaction term was non-significant and the main effect of UHR symptoms remained significant (see Table 6).

**Table 6:** Prediction of low psychosocial functioning (GAF score < 60) by UHR symptoms* and estimation of interaction with age effects.

Predictor	ß	SE	Wald (df)	p	Exp(ß)	95% CIs (Exp(ß))
Age	-.036	.041	.738 (1)	.390	.965	.890–1.047
any UHR symptom present	1.367	.421	10.554 (1)	.001	3.923	1.698–8.868
age * presence of any UHR	No interaction effect					

logistic regression analyses with method “backward” and “forward”.

* See Table 1 for a description of the UHR requirements

## 4. Discussion

In our sample of 22q11DS patients, we observed that UHR symptoms and criteria were uniformly common across age groups. Contrary to our first hypothesis, our results indicated no significant direct impact of age on the prevalence of UHR symptoms and criteria. The lack of age effect remained unchanged even when the analyses were run separately for perceptual and non-perceptual UHR symptoms. However, patients from the younger age groups were more likely to meet UHR criteria in the presence of UHR symptoms compared to the adult group. In addition, we observed that UHR symptoms were significantly associated with lower levels of functioning, regardless of age.

### 4.1 Effect of age on UHR symptoms and criteria

We observed that 38% of patients with 22q11DS presented with at least one UHR symptom, and 27% met at least any one UHR criterion. These numbers are within the midrange of those reported from other 22q11DS cohorts [6, 17, 19, 29]. Eight participants with UHR symptoms were not diagnosed with a UHR condition because the onset/worsening and the frequency criteria were not met, and 6 met the onset/worsening but not the frequency criterion. Compared to recent findings from the general population [20], 22q11DS was associated with a 3.8-fold increase of being diagnosed with UHR symptoms and a 20.8-fold increase of meeting UHR criteria. However and contrary to what has been observed in the general population [20], the presence of UHR symptoms was not modulated by age.

A more in-depth examination of the frequency of UHR symptoms in patients with 22q11DS across the different age groups revealed that their prevalence in affected children is comparable to previous estimates from the general population [19]. However, a clear difference was observed in older age groups, with a prevalence of 8% in adults from the general population [19] and 46% in adults with 22q11DS included in the present study. These findings may indicate that 22q11DS is associated with a persistence of manifestations that are commonly encountered during childhood [20, 29], rather than with the emergence of UHR symptoms during adolescence. Although this interpretation would require confirmation from longitudinal studies, it highlights the need to examine factors shown to predict the persistence of psychotic-like experiences in the general population also in 22q11DS cohorts. In particular, the persistence of self-reported hallucinations over time has been associated with phenomenological characteristics of these experiences (e.g. hostile tone), presence of comorbid disorders (e.g. anxiety and mood disorder) or environmental stressors (e.g. urbanicity and trauma) [30, 31]. The persistence of UHR symptoms in 22q11DS may also be influenced by an atypical development of specific cognitive processes that usually mature during the course of adolescence and have been involved in the pathway to psychosis. For example, meta-cognitive impairments have been linked to the presence of hallucinations in cognitive models of psychosis [31] and have been described in adolescents with 22q11DS [32, 33–35].

Although age was not associated with the prevalence of UHR symptoms, we did observe that being diagnosed with a UHR condition in the presence of UHR symptoms was more likely in the younger age groups compared to the adult group, indicating that both onset/worsening and frequency requirements for an UHR condition are more likely to be met at a younger age. This result might be driven by a recruitment bias in favour of younger patients being more frequently presented by their parents. Thus, adult participants involved in this study might be more dependent on seeking help on their own accord and thus selected for their insight and willingness for treatment and less representative of the 22q11.2 population compared to younger participants. However, this recruitment bias is unlikely, as the majority of adults (14 out of 26 adults included in this study) had already been recruited into the Rome or Geneva longitudinal studies before the age of 18 years. Alternatively, this result also indicates that the maximum risk period for being diagnosed with an UHR condition occurs during late childhood/early adolescence in 22q11DS. It is also in line with previous studies reporting an early mean age-of-onset for psychotic disorders in 22q11DS [12, 18, 36]. Thus, as the recommended first-choice cognitive-behavioural psychotherapy for CHR patients [37] requires certain cognitive skills [38], early intervention strategies should be developed that are specifically tailored to children and young adolescents with cognitive impairments and, in some cases, mild intellectual disability. Indeed, the vast majority of early intervention trials in UHR patients excluded individuals under the age of 14 years and below an IQ of 70 [14, 15, 39].

### 4.2 Impact of UHR symptoms on functioning and modulation by age

We found that the presence of UHR symptoms was associated with a significantly reduced level of functioning. This result was expected and in line with evidence from the general population. Indeed, functional impairments have been related to clinician-assessed UHR symptoms in children and adolescents [20, 40, 41] and young adults samples [42, 43]. Although the role of UHR symptoms on functioning in 22q11DS patients has been examined previously [3, 5], this is the first study to show that 22q11DS patients with UHR symptoms have a worse functioning than those without. From a clinical perspective, these results confirm that UHR symptoms are a cause of distress in patients with 22q11DS and should therefore be the target of specific therapeutic interventions.

Interestingly, we did not find any significant interaction between age and UHR symptoms on functioning. This result differs from the aforementioned study in the general population showing a strong age effect, with a significant shift in the clinical significance of APS, in particular non-perceptive ones, and their UHR requirements from early to late adolescence [20].

### 4.3 Should UHR symptoms be considered as a trait-like dimension in 22q11DS?

Taken together, the results of the present study show a lack of age effect on the prevalence and clinical relevance of UHR symptoms in 22q11DS. This represents a substantial difference compared to what has been reported in the general population and indicates that UHR symptoms might best be considered as trait-like phenomena in patients with 22q11DS. This view is supported by the large genetic contribution to the aetiology of UHR symptoms or psychosis in 22q11DS compared to the more multifactorial, and greater environmental-related aetiology in other UHR populations. In line with this is also the reported more homogeneous profile of UHR symptoms in patients with 22q11DS compared to UHR patients without the deletion [3]. This view can also be considered as an extension of the “syndrome-specific” hypothesis [44, 45], proposing that risk for psychopathology in genetic syndromes is caused by different neurobiological factors and abnormal brain development that vary depending on the aetiology of each specific genetic condition.

### 4.4 Strengths and limitations

This is the first study to date that examined the contribution of age in the prevalence and clinical significance of UHR symptoms and criteria in 22q11DS. However, additional work remains to be conducted to fully explore this question. In particular, longitudinal studies should examine whether the risk of conversion from a UHR condition to full-blown psychosis is increased in certain age groups. The sample was recruited from two clinical departments considered to be international points of reference for the assessment and treatment of psychiatric disorders in 22q11DS and is therefore likely to be highly representative of the 22q11DS population. The double-site recruitment strategy can represent at the same time a limitation of the study, since patients were recruited in two different clinical settings with different recruitment strategies. Therefore, it cannot be excluded that the sample is affected by different attribution biases. A second limitation is due to the relatively small sample size, which prevented a more detailed analysis of the interactions between the variables of interest. However, this limitation should be considered in light of the low prevalence of this genetic syndrome that makes the recruitment of large samples difficult. A second limitation is that the age effect findings could be influenced by an age of ascertainment bias. Indeed, parents of younger children who are already demonstrating psychotic symptoms may have been more likely to participate. Yet, this would have made a replication of the community findings of a higher prevalence of APS/BLIPS in children and young adolescents [19] more likely and does not explain the equally high prevalence in adult patients.

### 4.5 Conclusions

In conclusion, our data suggest that the risk of meeting UHR criteria is especially increased in late childhood and early adolescence in 22q11DS, although we observed that age did not significantly influence the prevalence or clinical significance of UHR symptoms in 22q11DS. This indicates the trait- rather than state character of these symptomatic risk markers for psychosis in this genetic high-risk population. Research in early interventions for psychosis is thus needed in 22q11DS in order to develop effective treatment strategies and reduce the long-term disability associated with this condition. Moreover, these results represents a confirmation that 22q11DS is of considerable interest to research on the genetic mechanisms involved in the development of schizophrenia-spectrum and cognitive-related disorders [46].

## Supporting information

S1 Fig Interaction of age and presence of UHR symptoms on UHR status. (DOCX)

S1 Table Baseline comparison between the two cohorts. (DOCX)
