# Supplementary figures and images for: No age effect in the prevalence and clinical significance of ultra-high risk symptoms and criteria for psychosis in 22q11 deletion syndrome: Confirmation of the genetically driven risk for psychosis?
Source: PLoS One. 2017 Apr 13;12(4):e0174797. doi: 10.1371/journal.pone.0174797 (PMC5390987; doi:10.1371/journal.pone.0174797)

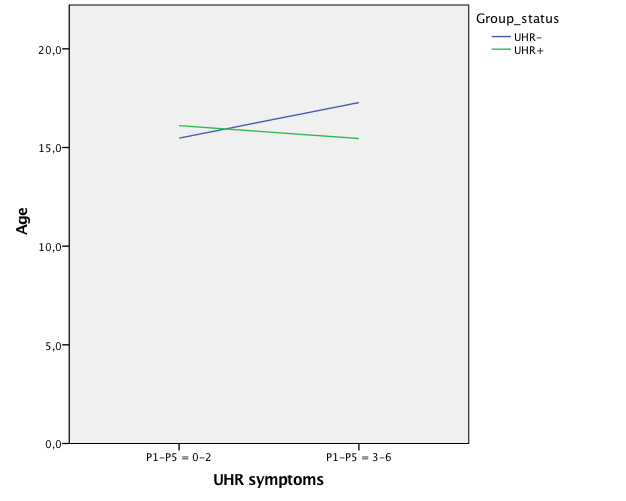


Supplementary Figure 1: Interaction of age and presence of UHR symptoms on UHR status

Supplement: S1 Fig — (DOCX) [file pone.0174797.s001.docx]
