# Supplementary material for: No age effect in the prevalence and clinical significance of ultra-high risk symptoms and criteria for psychosis in 22q11 deletion syndrome: Confirmation of the genetically driven risk for psychosis?
Source: PLoS One. 2017 Apr 13;12(4):e0174797. doi: 10.1371/journal.pone.0174797 (PMC5390987; doi:10.1371/journal.pone.0174797)
Supplement: S1 Table — (DOCX) [file pone.0174797.s002.docx]

Supplementary Table S1: Baseline comparison between the two cohorts. If not otherwise specified, mean (sd) are provided.

|  | **Rome (n = 34)** | **Geneva (n = 77)** | **Group comparison** |
| --- | --- | --- | --- |
| Age | 14.85 (4.92) | 16.01 (4.61) | t(109)=1.141, p=0.256 |
| Gender (%; females) | 16 (47.1) | 43 (55.8) | χ^2^(1)=0.982, p=0.576 |
| FSIQ | 84.13 (11.37) | 71.71 (10.78) | t(109)=-5.307, p < 0.001 |
| BLIPS (%) | 2 (5.8) | 1 (1.3) | χ^2^(1)=2.457, p=0.177 |
| APS (%) | 7 (20.6) | 15 (19.5) | χ^2^(1)=0.319, p=0.375 |
| GRFD (%) | 0 (0) | 11 (14.3) | χ^2^(1)=0.452, p=0.026 |
| Any DSM diagnosis (%; yes) | 23 (67.6) | 50 (64.9) | χ^2^(1)=2.17, p=0.105 |

FSIQ: full scale IQ; BLIPS: brief limited intermittent psychotic symptoms; APS: Attenuated psychotic symptoms; GRFD: generic risk + functional decline
